# Assessing the roles of absorption capacity in technological spillovers and economic growth nexus

**DOI:** 10.1371/journal.pone.0277651

**Published:** 2022-12-30

**Authors:** Mirajul Haq, Shahzad Hussain, Baber Amin

**Affiliations:** 1 International Institute of Islamic Economics (IIIE), International Islamic University Islamabad, Islamabad, Pakistan; 2 Department of Economics, Government College University Lahore, Lahore, Pakistan; Hainan University, CHINA

## Abstract

The paper aims to empirically assess the effects of technological spillovers on economic growth and to examine the roles of host country absorptive capacity. The empirical analysis was carried out at the country level on a panel of five Asian countries covering the period from 1972 to 2018. As the variable of interest (technological spillovers) and mediator variable (absorptive capacity) are captured with a variety of indicators, hence two empirical models are estimated with different specifications. The study’s findings indicate that technological spillovers through all three channels have a positive effect on economic and TFP growth. Touching on the role of absorptive capacity in technological spillovers and economic growth nexus, study findings reveal that the human capital of the sample countries has no significant role to absorbed imported technology in the growth process of the host country. However, the empirical indication illustrates that a country holding comparatively more domestic R&D expenditure yields the potential gain of technological spillovers in economic growth.

## 1. Introduction

In the mid-1980s, with the advent of endogenous growth theory, a large segment of economic literature has an eternizing interest in the relationship between international trade and economic growth. Broadly, the endogenous growth framework focused on two roles of international trade in the growth process. Models closer to the Neo-Classical view [1–7] try to explain the role of international trade in terms of human capital accumulation. While models based on the creative destruction idea [8–13] which strive to explain the role of international trade in the growth process in terms of innovation, technological improvement, and its transfer.

Studies based on the Schumpeterian approach suggest that international trade; affects economic growth in three ways. Firstly, international trade expands varieties of new products. [9] for example, highlights that an increase in knowledge rests on the introduction of a greater variety of goods and international trade plays a positive role in this connection. [14] argued that international trade in capital goods raises the market size for new product varieties. [15, 16] argued that trade openness is beneficial for introducing new varieties because it provides access to a broader base of technical knowledge, reducing innovation costs.

Similarly, the innovation-based-growth model of [11] argued that international trade provides opportunities for innovation and consequently leads to technological improvements. Secondly, international trade provides access to foreign intermediate inputs; e.g., [9] argued that international trade enables countries to import intermediate inputs from abroad that are not invented domestically. Thirdly, international trade facilitates the diffusion of global knowledge. For instance, [17] define that “international trade in intermediate goods is the main channel of international knowledge spillovers” and support the idea that knowledge diffused through trade seemed to raise domestic productivity.

Several studies suggest that, like international trade, embodied and disembodied technology spillovers are closely tied to FDI. Despite some conflicting evidence [18–20] numerous studies have suggested that FDI is the one central transmission channel through which technology spillovers across borders [21, 22]. The majority of these studies indicate that FDI contributes to international technology diffusion if Multinational Corporations transfer technology to their foreign associates and indirectly as the technologies employed by associates are diffused to local firms in the importing country. Most of the empirical insights came with the findings that trade and FDI are the two primary channels through which technology diffuses across borders.

Another vital contribution to this strand of literature is the analysis of a country’s absorption capacity that is coped through international knowledge. However, the advocates of outer-oriented regimes designate some conditions for dynamic gains of the liberalised regime, such as technological progress and transmission. For instance, they endorse that the country’s potential gain of liberalize trade regimes through technological diffusion revolves around its capacity to observe and adroitly utilize foreign knowledge and technology in its industrialization and economic growth process. In this context, recently, a group of empirical insights have underlined the importance of imported country absorption capacity [19, 23–27]. These studies have suggested and analyzed different components of absorptive capacity. Some have emphasized investment in education and human capital [19, 28] while others are more specific and emphasized investment in R&D [21, 29] and technological infrastructure [30, 31]. There are also studies that define absorption capacity in terms of social infrastructure, national orientation, and provisions of government incentives.

Studies that have analyzed the contribution of absorptive capacity, especially those that emphasised accumulation of human capital and investment in R&D, have two contrasting views about absorptive capacity. On the one hand [7] for instance argued that “the effective cost of innovation and technology adoption are higher when a country is further away from the world technology frontier”. On the other hand, [32–34] argued, “a country further behind the world technology frontier then every successful technology adoption leads to a greater improvement in the national technology level.” The indefiniteness in the studies on the subject is the basic motivation to carry this study. In this context, this study examines the impact of technological spillover on economic growth and investigates the role of absorption capacity. To come across this objective, we attempt to response some key queries. Firstly, by what means technological spillovers pose its impact on economic growth of the importing country. Secondly, by what means technological spillovers affect TFP growth of the importing country. Thirdly, in what manner an importing country’s absorption capacity play a role in the potential utilization of imported technology in its growth process.

Unlike existing empirical insights, this study is an addition to the existing studies on the subject by taking into account separately two outcome variables (i.e., economic growth, TFP growth). Moreover, absorptive capacity of the importing country is captured with two different proxies (i.e., human capital, domestic R&D). In this context, two empirical models are formulated. First, empirical model is formulated in order to examine the impact of technological spillovers on economic growth. The second model is formulated to examine the impact of technological spillovers on TFP growth. In both models, the role of importing country’s absorptive capacity have been investigated using two different proxies.

The empirical models are estimated using data set of five Asian countries spanning from 1972 to 2016 which are **Bangladesh, China, India, Pakistan and Sri Lanka**. Following reasons my justify why we limit our analysis to five regional Asian countries. Firstly, we select countries that heavily reliance on imported technology during the period under investigation. Secondly, during the sample period having replaced closed and import substitution trade polices with liberalized and export promotion strategies these regional countries passed through a paradigm shift. Thirdly, most of the sample countries have shown impressive economic growth during the period. The rest of the paper is organized as follows. Section 2 presents methodology that covers empirical model, definition and construction of variables, data source and estimation techniques. Section 3 discusses estimated results and its interpretations. Finally, section 4 offers some concluding comments.

## 2. Methodology

The methodology section contains four subsections. The subsection 2.1 presents empirical models, 2.2 shows the definition and construction of variables under consideration. Whereas 2.3 presents data and data sources. Finally, subsection 2.4 refers to estimation techniques.

### 2.1 Empirical models

In order to examine the technological spillover effect on economic growth and to investigate the role of absorption capacity in this nexus, we developed two different empirical models. These models are mainly formulated on the basis of the proposed models of [10, 14, 17, 27, 35–37].

#### Empirical model 1

A number of empirical studies, [10, 17, 24, 29, 34, 38–40] argue that a country can reap the potential benefits of imported technology in their growth process only when it possesses sufficient capacity to absorb it into its production process. Moreover, Dahlman and Nelson (1995) defines absorption capacity is the ability to adopt and implement the advance technologies and related application of developed countries. In this context, we structure an empirical model in order to investigate the role of the host country’s absorptive capacity in growth and technological spillover nexus. Ruminate the Hicks natural production function as follows.

$$Y_{t}=A_{t}K_{t}^{\beta }L_{t}^{1-\beta }$$ (1)

Where *Y*_*t*_ is output production, *K*_*t*_ is physical capital formation, and *L*_*t*_ is labor force. Consider [17] that technology is determined within the model (endogenously) and its level merely revolves around both domestic R&D and foreign R&D variables. Hence, the equation for technological level can be rewritten as;

$$A_{t}=DRD_{t}^{\alpha_{1}},FRD_{t}^{\alpha_{2}}$$ (2)

Taking log and adding intercept in Eq 2 will take the shape as,

$$lnA_{t}=\alpha_{0}+\alpha_{1}DRD_{t}+\alpha_{2}FRD_{t}$$ (3)

Where, *DRD*_*t*_ are domestic R&D variables and *FRD*_*t*_ is foreign R&D variables which affects the technological spillover effect on economic growth and absorptive capacity. Taking intensive form of Eq (1) takes the form;

$$y_{t}=A_{t}k_{t}^{\beta }$$ (4)

Taking log of the Eq (4) takes the following form;

$$lny_{t}=lnA_{t}+\beta lnk_{t}$$ (5)

Putting value of *lnA*_*t*_ from Eq (3)

$$lny_{t}=\alpha_{0}+\alpha_{1}lnDRD_{t}+\alpha_{2}lnFRD_{t}+\beta lnk_{t}$$ (6)

In Eq (6), the domestic *DRD*_*t*_ variables and foreign *FRD*_*t*_ variables are presented in weighted form as used by [17]. Whereas, the weighted FRD_t_ stock is proxy of technological spillover in manufacturing sector. In this regard, the weighted stock of both domestic and foreign R&D coefficients demonstrates the technological spillover. As the absorption of technology/knowledge spillover mainly depends upon country, human capital, domestic R&D effort, trade openness and intensity of technical labor force in country’s manufacturing sector. Hence, in this study we capture absorption capacity of the host country with three different proxies namely level of human capital, domestic R&D expenditure, and trade openness. Incorporating theses proxies and shaping in panel data structure, Eq (6) takes the following form;

$$lny_{it}=\alpha_{0}+\alpha_{1}lnDRD_{it}+\alpha_{2}\left(lnFRD_{it}*HC_{it}\right)+\alpha_{3}HC_{it}+\alpha_{4}\left(FRD_{it}*DRD_{it}\right)+\alpha_{5}TO_{it}+\alpha_{6}\left(FRD_{it}*TO_{it}\right)+\varepsilon_{it}$$ (7)

In Eq (7) human capital *HC*_*it*_ domestic R&D capital stock *DRD*_*it*_, and trade openness *TO*_*it*_ are different proxies of absorptive capacity. The slope parameters (*α*_2_, *α*_4_, *α*_6_) shows foreign R&D spillover which conditional to different proxies of absorptive capacity. Eq 7 is our first empirical model through which we will assess the impact of technological spillover on economic growth. In addition, the interactive terms of foreign R&D and different proxies of absorptive capacity will assess the role of absorptive capacity in technological spillover and economic growth nexus.

#### Empirical model 2

Our second empirical model is based on [10, 17, 24, 41] in which they modeled international technological spillover in the endogenous growth framework. These studies have demonstrated the effect of trade liberalization on domestic technological change, whereas the effect on domestic technology change is captured with change in total factors productivity (TFP). Like first model, we assume the Hicks-neutral production function with constant return to scale.

$$Y_{t}=A_{t}K_{t}^{\alpha }L_{t}^{1-\alpha }$$ (8)

Where Y_t_, K_t_ and L_t_ represents total production, physical capital stock and labor respectively. Taking log on both sides, Eq (8) takes the form;

$$lnY_{t}=lnA_{t}+\alpha lnK_{t}+\left(1-\alpha \right)lnL_{t}$$ (9)

Eq (9) shows that along with conventional factors that labor and capital, total factor productivity (TFP) is one of the key factors that explain economic growth as indicated in received literature, including [14, 17, 27, 29, 35–37]. Taking *lnA*_*t*_ as explained variable, Eq (9) takes the following form;

$$lnA_{t}=lnY_{t}-\alpha lnK_{t}-\left(1-\alpha \right)lnL_{t}$$ (10)

Eq (10) measures TFP, which is obtained by subtracting the contribution of conventional factors capital and labor from total output. As our interest is to investigate the impact of technological spillover on TFP, and therefore on economic growth. Hence, following the Schumpeterian growth framework [11, 14, 41], we capture the impact of imported technology on economic growth through its effects on TFP. By incorporating technological diffusion. Eq (10) will be extended as follows;

$$lnA_{it}=lnY_{it}-\alpha lnK_{it}-\left(1-\alpha \right)lnL_{it}+\;lnRD_{it}^{d}+lnRD_{it}^{f}+lnX_{it}$$ (11)

Where subscript *i* = 1, … …, *n* denote cross sectional unit and *t* = 1, … …, *n* denotes time dimension. Eq (11) indicates that total factor productivity *A*_*it*_ depends on domestic R&D ($RD_{it}^{d}$). This exhibits that a country which tends to rotates more resources towards R&D sector are more likely to upturn its TFP. ${RD}_{it}^{f}$ is foreign R&D intensity which is the indicator of technological diffusion. [24] argued that the country which has more spillovers of foreign R&D can acquire higher growth of TFP. Finally, *X*_*it*_ is the vector of imported technological goods. Eq 11 is our second empirical model through which we want to examine the impact of technological spillovers on TFP.

### 2.2 Definition and construction of variables

This section of the study discusses the definition and construction of the variables under consideration. The subsequent subsections present the definition and construction of dependent and independent variables, respectively.

#### Dependent variables

We have two empirical models, hence two dependent variables, namely, economic growth (growth of GDP) and Total Factor Productivity (TFP). Our first dependent variable is economic growth (Y_it_), which is captured with an annual percentage growth of GDP. Total Factor Productivity (*A*_*it*_) is our second dependent variable which is calculated as, *lnA*_*t*_ = *lnY*_*t*_ − *αlnK*_*t*_ − (1 − *α*)*lnL*_*t*_.

Here *A*_*t*_ is TFP, *Y*_*t*_ is output production, while *K*_*t*_ and *L*_*t*_ are capital stock and labor force respectively.

#### Explanatory variables

Among explanatory variables, technological spillovers (FRD_it_) is our variable of interest. The technological spillover is proxy with the inflow of FDI (*FDI*_*it*_) to host country and its imports of machinery and transport equipment (*IMPC*_*it*_). Following [17], with these channels, technological spillovers is calculated as follows;

$${\text{FRD}}_{\text{it}}=\frac{{\text{FDI}}_{\text{it}}}{\sum {\text{FDI}}_{\text{it}}}{\;*\;\text{DRD}}_{\text{it}}\;\text{the}\;\text{inflow}\;\text{of}\;\text{technological}\;\text{spillovers}\;\text{throug}\;\text{FDI}\;\text{channel}$$

Here *FRD*_*it*_ is weighted foreign R&D expenditure and *FDI*_*it*_ is foreign direct investment inflow, *DRD*_*it*_ is domestic R&D capital stock. Similarly, by taking into account the imports channel, *FRD*_*it*_ can be calculated using bilateral import share multiplied R&D intensity.

$$FRD_{it}=\frac{{\text{BI}}_{\text{it}}}{\sum {\text{BI}}_{\text{it}}}*RDINT_{it}$$

Where, *BI*_*it*_ is a bilateral import of technological goods (machinery and transport equipment) and Σ*BI*_*it*_ is total imports of goods and services. Here *RDINT*_*it*_ denotes the R&D intensity which is expressed as gross domestic R&D expenditures as percentage of GDP.

Our second variable of interest is absorptive capacity (AC_it_). In general, it is believed that the potential growth effectiveness of imported technology depends on imported country absorption capacity that cope international knowledge/technology in its production process. Received literature on the subject [19, 28, 29, 42, 43] offers different proxies to measure absorption capacity of imported countries containing human capital stock, R&D expenditure. To capture the impact of absorptive capacity more rigorously, we used both proxies of absorptive capacity in our analysis. Among these, the domestic R&D stock (*DRD*_*it*_), is capture with the expenditure on the research and development sector in the country territory within a specific time period. These expenditures are carried out on research institutes, universities, resident companies, and government laboratories, within a country territory. The domestic R&D stock is constructed by using the Perpetual Inventory Method (PIM) which allows the depreciation rate (we set depreciation rate at 5 percent level for calculate the domestic R&D stocks).${DRD}_{it}=\left(1-\delta \right){DRD}_{it-1}+I_{it}^{RD}$. Where, *DRD*_*it*_ is domestic R&D stock, *δ* is deprecation rate and $I_{it}^{RD}$ is total R&D expenditure in the economy.

The human capital stock (*HC*_*it*_) is the composite index, which is developed on the bases of years of schooling and return on education that available at Penn World Table 9.0. Similarly, trade and FDI openness are measured through trade to GDP and FDI to GDP ratio respectively. The control variables are physical capital stock (*PhyC*_*it*_) is measured by taking the difference between gross capital formation and consumption on fixed capital (depreciation or capital consumption). Perpetual Inventory Methodology is applied for calculating the net capital formation, which was developed by [44].

$$K_{it+1}=I_{it}+\left(1-d\right)K_{it}$$

${K_{it}}_{+1}$ is net capital stock in country *i* at period *t* + 1, *I*_*it*_ is gross capital stock, and ′*d*′ is deprecation rate, and *K*_*it*_ is capital stock at current period *t*. Population growth (*POP*_*it*_) is the annual percentage growth of population, similarly inflation (*INF*_*it*_) is the annual percentage change in the consumer price index. Labor force (*LF*_*it*_) is measured as working age population as a percentage of the total population.

### 2.3 Data and data sources

As the study was carried out for a panel of five Asian countries over the period over the period 1972–2018, the data of the sample courtiers is taken from secondary sources, including, Penn World Table (PWT), Latest Vision 9.0, UN Comtrade Statistics (UNCTAD); UNESCO Data Set; Ibero American Science and Technology Indicator Network (RICYT); OECD Industrial and Services Statistics-Structural Analysis (STAN) Databases-R&D expenditure in industry.

### 2.4 Estimation techniques

Breusch-Pagan’s (1979) test was employed to test whether the intercept values are the same across cross-sections or not. In both empirical models and all specifications the null hypothesis of the Breusch-Pagan test *δ*^2^ = 0 was accepted, indicating that intercept values are the same across cross-sections. This suggests that Pooled OLS estimation is the appropriate estimation technique. Hence, the empirical estimations were carried out through Pooled OLS.

## 3. Empirical results

As the study aims to explore the technological spillover effects on economic growth and the role of absorptive capacity by following the empirical literature on the subject [17, 35, 45], we approach towards empirical analysis with two different empirical models. Sections 3.1 and 3.2 present estimated results of our empirical model one (Eq 7) and two (Eq 11), respectively.

### 3.1 Estimated results of empirical model 1

Following Table 1 presents the estimated results of our empirical model 1 (Eq 7), whereas the dependent variable is economic growth (GDP growth).

**Table 1:** Estimated results (dependent variable is GDP growth).

Variables	Spec. 1	Spec. 2	Spec. 3	Spec. 4	Spec. 5	Spec. 6
**PhC_it_**	4.631*** (3.81)	8.052** (2.69)	4.641*** (8.21)	6.266*** (8.61)	6.333*** (8.22)	5.681*** (7.91)
**HC_it_**	1.696** (2.40)	2.041* (1.85)	0.796*** (4.97)	0.429** (2.03)	6.464*** (4.15)	3.903*** (4.33)
**INF_it_**	-0.011 (-1.80)	-0.003 (-0.26)	-0.001 (-0.14)	0.023 (1.50)	-0.002 (-0.24)	-0.032** (-1.76)
**LF_it_**	0.624*** (7.01)	0.454*** (7.94)	-----	0.0725 (0.74)	0.062*** (8.38)	-----
**POP_it_**	-----	----	-0.157 (-1.28)	-----	-----	-0.325* (-1.92)
**TO_it_**	1.016*** (3.59)	-----	-----	1.240*** (4.83)	-------	----
**FDI_it_**	-----	0.263*** (2.61)	-----	----	0.368*** (6.31)	-------
**IMPC_it_**	-----	-----	0.226*** (7.02)	-----	----	0.242*** (0.52)
**DRD_it_**	-----	------	-----	0.397*** (4.92)	0.429*** (8.13)	0.373*** (4.68)
(**TO** * **HC**)_**it**_	-0.281** (-1.94)	----	-----	----	------	------
(**FDI** * **HC**)_**it**_	----	-0.087* (-1.89)	-----	----	-----	----
(**IMPC** * **HC**)_**it**_	----	----	-0.097*** (-6.43)	-----	-----	-----
(**TO** * **DRD**)_**it**_	-----	----	-----	3.437*** (5.68)	-----	------
(**FDI** * **DRD**)_**it**_	------	-----	-----	------	1.769*** (5.30)	------
(**IMPC** * **DRD**)_**it**_	-------	-------	------	------	-------	2.581*** (7.31)
Obs.	161	161	161	124	124	124
F-Stat.	184.02*** (0.000)	176.28*** (0.000)	110.20*** (0.000)	126.17*** (0.000)	137.24*** (0.000)	122.04*** (0.000)
R^2^	0.96	0.81	0.85	0.81	0.92	0.83
B.P-test	0.46 (0.497)	0.45 (0.501)	0.52 (0.348)	0.72 (0.462)	0.78 (0.474)	0.84 (0.635)

Note: Numbers in parentheses are values of t-statistic.

*, **, *** shows level of significance at 10, 5 and 1 percent respectively.

In specification 1 (column 2), the variable of interest (technological spillover) is captured through trade openness (LTO_it_), where the role of absorptive capacity is capture through human capital (*HC*_*it*_). The role of absorption capacity (human capital) in technological spillovers and economic growth nexus is captured with an interactive term of human capital with trade openness (HC * TO)_it_. The estimated results presented in Table 1 indicate that trade openness (TO_it_) carries a significantly positive coefficient as indicated in received literature. The trade openness (TO_it_) has a significant and positive impact on economic growth, which implies that trade openness can serve economic growth through spillover channels in selected countries such that our fitted values are in line with the findings of [43, 46]. Similarly, the coefficient of human capitalsubscript base, open paren HC, the impact of human capital on economic growth in selected countries. Estimated results indicate that investment in human capital raises the output level by improving labor efficiency and ultimately promoting economic growth by enhancing the absorptive ability to learn advanced technology and knowledge. The interactive term of trade openness and human capital (*HC* * *TO*)_*it*_ enters the model significantly and with a negative sign, which indicates that sample countries have less absorptive capacity to cope into foreign technology. This indicates that production units in the host country have less ability to install and replace imported (foreign) technology.

Among control variables, physical capital (*PhyC*_*it*_) holds positive coefficient that is statistically significant, indicates that physical capital is one of the core variables that explain economic growth in the sample country. The coefficient of labor force (*LLF*_*it*_) indicates a positive and significant impact on economic growth in sample countries. The labor force channel is linked with the elasticity of the labor force which shows that a one percent increase in labor force is translated into a 1.05 percent increase in sample countries economic growth. The inflation rate (*INF*_*it*_) does not exert any significant effect on economic growth in our first specification.

In speciation 2 (column 3), knowledge spillover is acquired through FDI inflow (FDI_it_), and the absorptive capacity is still human capital, hence the role of absorption capacity is capture with the interactive term of foreign direct investment and human capital (*FDI* * *HC*)_*it*_. Estimated results indicate that net inflow of FDI signify its role in the growth process of sample countries as (FDI_it_) enters in the model positively, which is statistically significant. Similarly, human capital (HC_it_) holds positive and significant coefficient. However, the interactive term (HC * FDI)_it_ enters the model negatively that is statistically significant. The one possible justification is emerging status of the two largest economies (China, and India) in our sample. For instance, [21, 47] argued that due to larger inequality in China’s provinces involve less absorptive ability and erudition of new technology which embodied in FDI. In addition, anticipated elasticity of labor force is larger than FDI, which shows that output growth rise through labor force efficiency is more effective than FDI inflow. This may be due to the reason that mostly selected countries are producing labor abundant products and therefore exporting labor intensive products. It is sensible for such developing countries (having large share of world population) to produce labor intensive products with output elasticity of 0.454. Our findings are consistent with the findings [43].

Specification 3 (column 4) shows the estimated results of our empirical model 1, whereas technological spillover is capture through technology import (IMPC_it_) and the role of absorptive ability is captured through interactive term (HC * IMPC)_it_. The coefficient of technology imports is positive and statistically significant. The results demonstrate that imported technology play a significant role in the growth process of the sample countries. However, the interactive term (HC * IMP)_it_ holds negative sign, which is statistically significant. Results indicate that sample countries have less capacity to absorbed imported technology in its production process.

In model 4 (column 5), the technological spillovers are proxy with trade openness (TO_it_), whereas the absorptive capacity of host country is captured through domestic R&D expenditure (DRD_it_).

The estimated coefficient of domestic R&D carries a positive sign which is significant at one percent level, which indicates that domestic R&D has positive impacts on economic growth in selected countries. To analyze the role of domestic R&D expenditure we add an interactive term of trade openness and domestic R&D (TO * DRD)_it_. The interaction term enters the model positively that is statistically significant. Result reveals that domestic R&D play a vital role in the technological spillovers and economic growth nexus. Results indicates that the higher the domestic R&D expenditure, the greater would be the potential gain of imported technology in growth process.

In model 5 (column 6), trade openness is replaced with FDI inflow (FDI_it_), whereas the absorptive capacity of host country is remained domestic R&D expenditure (DRD_it_). Hence, the role of absorptive capacity is captured through interactive term of FDI and domestic R&D (FDI * DRD)_it_. Similar to previous case, the interaction term holds positive sign which is statistically significant. Results exposes that country having holds relatively more domestic R&D should gain more output of technological spillovers through FDI in its growth process. In model 6 (column 7), the technology spillovers are captured through imports of machinery and transport equipment (IMPC_it_), which enters the model positively and statistically significant. The absorptive capacity is still capture through domestic R&D expenditure (DRD_it_). The role of absorptive capacity is captured through interactive term of imported technology and domestic R&D (IMPC * DRD)_it_. The interactive term enters the model positively and statistically significant. Like trade openness and FDI this indicates that countries, which holds more domestic R&D should take more benefits of imported technology in its growth process. To be exact more domestic R&D of host country results a potential utilization of imported technology in its production process that in turn enhancing growth of host country. The control variables physical capital, human capital, inflation holds same results in all specifications. The higher values of F-statistic in all specifications indicates that models are fit. In addition, the R^2^ value is greater than 0.8 in all specifications, indicates that more than 80 percent variation in dependent variable is explain by explanatory variables.

### 3.2 Estimated results of empirical model 2

Table 2 presents estimated results of empirical model 2 (Eq 11), whereas the dependent variable is TFP growth. As the null hypothesis of [48] test *δ*^2^ = 0 is accepted, hence the empirical model is estimated through pooled OLS.

**Table 2:** Estimated results (dependent variable is TFP).

Variables	Spec. 1	Spec. 2	Spec. 3	Spec. 4	Spec. 5	Spec. 6
**HC_it_**	0.057*** (2.99)	0.014*** (5.31)	0.127 *** (16.42)	0.007*** (3.05)	0.324*** (2.89)	0.141*** (14.24)
**POP**_**it**t_	0.03** (2.16)	0.002 (1.60)	0.005 (1.04)	0.002 (1.49)	0.006 (0.97)	0.002** (4.41)
**LF_it_**	------	0.002*** (3.46)	0.041* (1.78)	.0006 (0.50)	----	-----
**TO_it_**	0.021*** (2.75)	-----	-----	0.235*** (3.99)	-----	----
**FDI_it_**	-----	0.016*** (3.96)	-----	----	0.126*** (4.71)	-----
**IMPC_it_**	-----	-----	0.020*** (11.88)	-----	----	0.116*** (5.46)
**DRD_it_**	-----	------	-----	0.038** (2.20)	0.262*** (1.04)	0.321*** (4.51)
**INDAV_it_**	-------	-------	-----	------	------	-------
(**TO** * **HC**)_**it**_	0.026 (0.15)	----	-----	----	----	------
(**FDI** * **HC**)_**it**_	----	0.035 (0.35)	-----	----	-----	-----
(**IMPC** * **HC**)_**it**_	----	----	0.016 (0.31)	-----	-----	------
(**TO** * **DRD**)_**it**_	-----	-------	-----	0.417*** (3.27)	-------	------
(**FDI** * **DRD**)_**it**_	------	-------	-------	--------	0.714*** (4.810)	------
(**IMPC** * **DRD**)_**it**_	------	------	-------	-------	------	0.374*** (3.88)
Obs.	161	161	161	124	124	124
F-Stat	124.02*** (0.000)	123.15*** (0.000)	181.22*** (0.000)	113.12*** (0.0102)	112.18*** (0.000)	96.27*** (0.000)
R^2^	0.83	0.81	0.83	0.88	0.91	0.92
B.P-Test	1.89 (0.171)	0.86 (0.544)	1.82 (0.481)	1.54 (0.474)	0.93 (0.846)	1.53 (0.824)

Note: Numbers in parentheses are values of t-statistic.

*, **, *** shows level of significance at 10, 5 and 1 percent respectively.

Similar to empirical model 1, the technological spillovers are captured with trade openness (TO_it_), FDI inflow (FDI_it_) and import of machinery and transport equipment IMPC_it_. The absorptive capacity of host country is captured with host country human capital stock HC_it_ and domestic R&D (*DRD*_*it*_). In model 1 (column 2), the estimated results indicates that human capital (*HC*_*it*_) have a positive effect on TFP of the sample countries, similarly our variable of interest technology spillovers that captured with trade openness (TO_it_) carries a carries a significantly positive coefficient. The role of absorption capacity (human capital) in technological spillovers and TFP growth nexus is captured with interactive term of human capital with trade openness (HC * TO)_it_. The interactive term of trade openness and human capital (HC * TO)_it_ enters the model positively but statistically insignificant, which indicates that sample countries human capital have less absorptive capacity to cope into foreign technology.

In speciation 2 (column 3), technology spillover is acquired through FDI inflow (FDI_it_), and the absorptive capacity is still human capital, hence the role of absorption capacity is capture with the interactive term of foreign direct investment and human capital (FDI * HC)_it_. Estimated results indicate that FDI inflow holds a positive sign which is statistically significant. This signifies the role of FDI in TFP growth of the sample countries. However, similar to specification 1, the interactive term (HC * FDI)_it_ enters the model positively, which is statistically insignificant. This implies that human capital of sample countries has less capacity to absorb foreign technology in its TFP growth process having diffuse through FDI inflow.

Specification 3 (column 4) shows the estimated results of our empirical model 2, whereas technological spillover is capture through technology import (IMPC_it_) and the role of absorptive ability is captured through interactive term (HC * IMPC)_it_. The coefficient of technology imports is positive and statistically significant. This implies that imported technology play a significant role in the TFP growth of the sample countries. However, the interactive term (HC * IMP)_it_ holds positive sign, which is statistically insignificant. Results indicates in sample countries, human capital cannot play any role in the technological spillovers and TFP growth of the sample countries.

Models 4, 5 and 6 give the results of the sensitivity analysis where domestic R&D expenditure (DRD_it_) is used as a proxy of absorptive capacity. However, the variables of interest (technological spillovers) are unchanged that trade openness, FDI, and imports of machinery and transport equipment are used as proxies of technological spillovers. In all three specification the estimated coefficients of domestic R&D carry a positive sign which is significant at one percent level, which indicates that domestic R&D has positive impacts on TFP growth in selected countries. To analyze the role of domestic R&D expenditure we add an interactive term of trade openness and domestic R&D (TO * DRD)_it_. The interaction term enters the model positively that is statistically significant. Result reveals that domestic R&D prove beneficial in the technological spillovers and TFP growth nexus. Results indicates that the higher the domestic R&D expenditure, the greater would be the potential gain of imported technology in the form of TFP growth. Similarly, in next two specifications (5 and 6) the interaction terms FDI and domestic R&D (FDI * DRD)_it_ and imported technology and domestic R&D (IMPC * DRD)_it_ holds positive signs that are statistically significant. Results exposes that country having holds relatively more domestic R&D should gain more benefits of technological spillovers through FDI and imports in its TFP growth process. The higher values of F-statistic in all specifications indicates that models are fit. Like empirical model, the R^2^ value is greater than 0.8 in all specifications, which indicates that more than 80 percent variation in dependent variable is explain by explanatory variables.

## 4. Conclusion

Technological progress is the mutually acceptable mechanism among economists to accumulate and sustain the economic growth of a country. Besides, at large, economists have a consensus that the only effective and compelling approach for developing countries to catch up with the global technological frontier is by dint of its exposure towards foreign technology. However, they endorse that the country’s potential gain from technological spillovers revolves around the country’s capacity to observe and adroitly utilize foreign knowledge and technology in its production and economic growth process. This study empirically assessed the impacts of technological spillovers on economic growth, have investigated the role of absorptive capacity. In this context, two empirical models have been formulated and estimated. The first empirical model is developed to examine the impact of technological spillovers on economic growth while investigating the absorptive capacity’s role. The second model is formulated to examine the effects of technological spillovers on TFP growth and to investigate the role of absorptive capacity. The findings of the study reveal that technological spillovers through all three channels pose positive effects on the economic and TFP growth of the sample countries. Touching on the role of absorptive capacity in technological spillovers and growth nexus, the findings of the study reveal that the human capital of the sample countries has no significant role to absorbed imported technology in the growth process of the host country. However, the empirical indication illustrates that country having held comparatively more domestic R&D expenditure yields the potential gain of technological spillovers in the process of economic and TFP growth.

These indications provide some policy implications about trade, FDI, and economic growth of the sample countries. First, as the results of our first two models indicates trade openness, and FDI have a positive impact on economic growth, and growth of TFP, hence the sample countries need to implement outer oriented regime in order to increase and sustain growth of TFP and GDP. Second, our findings support the import of technology for both GDP and TFP growth, which demands a trade policy that facilitates and encourages imports of technology. Third, an important policy guidance that the study seems to suggest is that the country’s absorption capacity (human capital, domestic R&D) needs to be improved in order to harvest the potential benefits of imported technology. Accordingly, government policies should be designed in such a way to maintain incentives for the accumulation of human capital and R&D.
